# Modulation of anti-cancer drug sensitivity through the regulation of mitochondrial activity by adenylate kinase 4

**DOI:** 10.1186/s13046-016-0322-2

**Published:** 2016-03-16

**Authors:** Koichi Fujisawa, Shuji Terai, Taro Takami, Naoki Yamamoto, Takahiro Yamasaki, Toshihiko Matsumoto, Kazuhito Yamaguchi, Yuji Owada, Hiroshi Nishina, Takafumi Noma, Isao Sakaida

**Affiliations:** Center for Regenerative Medicine, School of Medicine, Yamaguchi University, Ube, Japan; Department of Gastroenterology and Hepatology, School of Medicine, Yamaguchi University, Ube, Japan; Division of Gastroenterology and Hepatology, School of Medical and Dental Sciences, Niigata University, 1-757 Asahimachidori, Chuo-Ku, Niigata 951-8510 Japan; Department of Oncology and Laboratory Medicine, School of Medicine, Yamaguchi University, Ube, Japan; Department of Organ Anatomy, School of Medicine, Yamaguchi University, Ube, Japan; Department of Developmental and Regenerative Biology, Medical Research Institute, Tokyo Medical and Dental University, 1-5-45 Yushima, Bunkyo-ku, Tokyo, 113-8510 Japan; Department of Molecular Biology, Institute of Biomedical Sciences, Tokushima University School, Tokushima, Japan

**Keywords:** Adenylate kinase, Drug resistance, Energy metabolism, Flux analysis, Hypoxia, Metabolome, Mitochondria

## Abstract

**Background:**

Adenylate kinase is a key enzyme in the high-energy phosphoryl transfer reaction in living cells. An isoform of this enzyme, adenylate kinase 4 (AK4), is localized in the mitochondrial matrix and is believed to be involved in stress, drug resistance, malignant transformation in cancer, and ATP regulation. However, the molecular basis for the AK4 functions remained to be determined.

**Methods:**

HeLa cells were transiently transfected with an AK4 small interfering RNA (siRNA), an AK4 short hairpin RNA (shRNA) plasmid, a control shRNA plasmid, an AK4 expression vector, and a control expression vector to examine the effect of the AK4 expression on cell proliferation, sensitivity to anti-cancer drug, metabolome, gene expression, and mitochondrial activity.

**Results:**

AK4 knockdown cells treated with short hairpin RNA increased ATP production and showed greater sensitivity to hypoxia and anti-cancer drug, *cis*-diamminedichloro-platinum (II) (CDDP). Subcutaneous grafting AK4 knockdown cells into nude mice revealed that the grafted cells exhibited both slower proliferation and reduced the tumor sizes in response to CDDP. AK4 knockdown cell showed a increased oxygen consumption rate with FCCP treatment, while AK4 overexpression lowered it. Metabolome analysis showed the increased levels of the tricarboxylic acid cycle intermediates, fumarate and malate in AK4 knockdown cells, while AK4 overexpression lowered them. Electron microscopy detected the increased mitochondrial numbers in AK4 knockdown cells. Microarray analysis detected the increased gene expression of two key enzymes in TCA cycle, succinate dehydrogenase A (SDHA) and oxoglutarate dehydrogenease L (OGDHL), which are components of SDH complex and OGDH complex, supporting the metabolomic results.

**Conclusions:**

We found that AK4 was involved in hypoxia tolerance, resistance to anti-tumor drug, and the regulation of mitochondrial activity. These findings provide a new potential target for efficient anticancer therapies by controlling AK4 expression.

**Electronic supplementary material:**

The online version of this article (doi:10.1186/s13046-016-0322-2) contains supplementary material, which is available to authorized users.

## Background

Adenylate kinase (AK) is an enzyme that regulates adenine nucleotide metabolism and homeostasis in a wide range of organisms, by catalyzing the interconversion reaction: ATP + AMP ⇌ 2 ADP. Nine different kinds of human AK isozymes have been reported. AK1, AK5, AK7, AK8, and AK9 are localized in the cytoplasm, AK6 and AK9 in the nucleus, AK2 in the mitochondrial intermembrane space, and AK3 and AK4 in the mitochondrial matrix. AK1 deficiency causes a hematological abnormality in human [1]. AK2 is essential for growth in *D. melanogaster* [2] and a genetic AK2 deficiency in human causes reticular dysgenesis and sensorineural deafness [3, 4], indicating the important role of AK2 in hematopoietic differentiation as well as development of auditory organ. Until now, we have studied the structure and function of AK isozymes and reported that they play important roles in cellular energy metabolism [2, 5–8]. We also reported that the therapeutic efficacy of iron chelator, deferoxamine (DFO) for treating hepatocellular cancer [9]. During the study, we found that *AK4* gene expression was up-regulated by DFO administration, although the biological meaning remained unclear. The cDNAs encoding the human and mouse *AK4* gene have been previously cloned [8, 10], and its expression pattern has been extensively characterized in mouse tissues [5], where AK4 was detected in the mitochondrial matrix, but did not display any enzymatic activity. Subsequently, other group reported that inactive AK4 interacted with adenine nucleotide translocase (ANT) [11]. On the contrary, another group reported that AK4 was enzymatically active using AMP: GTP and AMP: ATP as its substrates [12]. Therefore, it is still controversial whether AK4 shows classical enzymatic activity or not. The discrepancy of enzymatic activity data seems according to differences in the assay systems employed. Additional functional study indicated that AK4 may be involved in oxidative stress response by showing it as one of the proteins up-regulated by the administration of four types of agents that exhibit hepatic toxicity including carbon tetrachloride [13]. We have previously reported the cell- and tissue-specific expression profile of AK4 in mouse tissues [5]. In addition, it was reported that nucleotide synthesis showed in-day fluctuation, and AK4 expression was rhythmic in murine liver [14]. Interestingly, an independent study found that lung cancers with high AK4 expression showed increased malignancy [15]. Moreover, it was reported that AK4 provided a valuable marker of cellular stress in HEK293 and HepG2 cell lines [16]. Recently, Lanning et al. found that AK4 was the key regulator of intracellular ATP levels by screening an RNA interference (RNAi) library targeting over 1000 nuclear DNA-encoded genes whose protein products localized to the mitochondria [17, 18]. However, the molecular basis for the regulation of AK4-mediated ATP levels remains unclear, and the mechanisms how AK4 plays a role in oxidative stress and malignant transformation and regulates the mitochondria have not been elucidated. To address these questions, we carried out both in vitro and in vivo studies to investigate the effects of AK4 on cell growth, mitochondrial activity, metabolome, and gene expression.

## Methods

### Cell culture and reagents

HeLa cells were confirmed as the same cell line registered in the Japanese Collection of Research Bioresources Cell Bank (JCRB). A549 cells were purchased form JCRB. For the hypoxia treatment, cells were cultured in an incubation chamber at 37 °C, with 5 % CO_2_ and 1 % O_2_.

### Animals

All experiments were carried out in accordance with the guidelines approved by the Committee on the Ethics of Animal Experiments at the University of Yamaguchi. All surgery was performed under sodium pentobarbital anesthesia, and every effort was made to minimize suffering. BALB/c athymic nude mice were sacrificed using an overdose of anesthetic.

### Cell growth and ATP measurement

Cell proliferation under normoxic conditions was measured by real-time cell analysis using modified 16-well plates (E-plate, Roche Diagnostics). Studies were conducted after incubating the plated cells at 37 °C for 30 min to allow cell attachment, in accordance with the manufacturer’s guidelines. The data were expressed as a cell index value (CI). Changes in cell proliferation under hypoxia were assessed using the CyQUANT® Cell Proliferation Assay kit (Life Technologies) according to the manufacturer’s instructions. The ATP concentration was measured by the CellTiter-Glo™ Luminescent Cell Viability Assay kit (Promega).

### Western blot analysis

Protein lysates were obtained by homogenizing tissues or cell pellets in sample buffer containing 62.5 mM Tris-HCl (pH 6.8), 4 % sodium dodecyl sulfate, 200 mM dithiothreitol, 10 % glycerol, and 0.001 % bromophenol blue at a ratio of 1:10 (w/v), followed by boiling. Western blot analysis was performed using purified polyclonal anti-human AK4 rabbit IgG and antibodies against β-actin (Sigma), α-tubulin (Sigma), phosphorylated 5΄ AMP-activated protein kinase (p-AMPK; Abcam), hypoxia inducible factor 1α (HIF1α; Cell Signalling), hexokinase 2 (HK2; Abcam), ATP5a (Abcam), and the voltage-dependent anion channel (VDAC; Abcam), which were purchased from the indicated suppliers. Subcellular fractionation was conducted using mitochondria isolation kit (Thermo Fisher Scientific)sccording to the manufacturer’s instruction.

### Immunohistochemistry

HeLa cells were cultured in chamber slides for 2 days, and then fixed in 4 % paraformaldehyde. Prior to immunohistochemical staining, endogenous peroxidase in the fixed tissue slices was blocked by treatment with fresh 0.3 % hydrogen peroxidase in methanol for 30 min at 4 °C. Blocked samples were incubated with antibodies overnight at 4 °C. After washing 3 times in phosphate-buffered saline (PBS), the sections were incubated with biotin-conjugated secondary antibody in PBS for 3 h at 20 °C. After 3 additional PBS washes, a peroxidase-anti-peroxidase complex and streptavidin were added and incubation was maintained at 20 °C. Positive reactions were developed for 5-10 min using Tris-HCl buffer containing hydrogen peroxidase and 3,3΄-diaminobenzidine. Normal rabbit serum (Vector Laboratories, Burlingame, CA, USA) was used as a negative control.

### Total RNA isolation

Total RNA was isolated from HeLa cells using TRIzol Reagent (Life Technologies)), according to the manufacturer’s instructions. RNA samples were quantified by an ND-1000 spectrophotometer (NanoDrop Technologies, Wilmington, DE).

### Expression and suppression of AK4

HeLa cells were transiently transfected with small interfering RNA (siRNA) or with and AK4 short hairpin RNA (shRNA) plasmid (TRCN37554), a control shRNA plasmid, a green fluorescent protein (GFP)-tagged AK4 expression vector (RG220572), and a control expression vector, purchased from Sigma-Aldrich, Tokyo, Japan or from Origene, Rockville, USA, respectively. The siRNA and shRNA sequences were as follows; AK4 siRNA #1, GUCAUUGAAUUAUACAAGATT; AK4 siRNA #2, CAUCUUUUCUAGUUGAAAUTT; AK4 shRNA, GCCAGGCTAAGACAGTACAAA. Silencer® Select Negative Control #1 siRNA and Control #2 siRNA were purchased from Life Technologies. HeLa cells transfected with shRNA were selected by puromycin. HeLa cells transfected with GFP-tagged AK4 expression vector or control vector were selected by flow cytometer and G418.

### Gene expression analysis by microarrays

The cRNA was amplified, labeled, and hybridized to a 60 K Agilent 60-mer oligomicroarray, according to the manufacturer’s instructions. All hybridized microarray slides were scanned by an Agilent scanner. Relative hybridization intensities and background hybridization values were calculated using Agilent Feature Extraction Software (9.5.1.1). G3 Human Gene Expression Microarray 8 × 60 K, v2, was used for analysis. The raw signal intensities of all samples were log2-transformed and normalized using the quantile algorithm of the ‘preprocessCore’ library package on Bioconductor software. We selected the probes, excluding the control probes, where the detection *P*-values of all samples were less than 0.01, and used these to identify differentially expressed genes. We then applied the Linear Models for Microarray Analysis (limma) package within the Bioconductor software. The results derived from this study have been deposited in the National Center for Biotechnology Information (NCBI) Gene Expression Omnibus and are accessible through the Gene Expression Omnibus (GEO) Series, Accession Number GSE61843 (http://www.ncbi.nlm.nih.gov/geo/query/acc.cgi?&acc = GSE61843).

### Measurement of oxygen consumption rate (OCR)

OCR measurements were performed using a Seahorse Biosciences XF96 Extracellular Flux Analyzer. HeLa cells were seeded at 10,000 cells/well in XF96 microplates (Seahorse Biosciences). After a 24-h incubation, the growth media were exchanged for XF Assay Medium (Seahorse Biosciences) supplemented with 25 mM glucose (Sigma-Aldrich). OCR measurements were made over 5-min periods following a 3-min mix period. HeLa cells were treated by sequential addition of 1 μg/mL oligomycin (Sigma-Aldrich), 300 nM carbonylcyanide-*p*-trifluoromethoxyphenylhydrazone (FCCP; Sigma-Aldrich), and 2 μM rotenone (MP Biomedicals). The spare respiratory capacity and coupling efficiency were calculated according to the Seahorse Bioscience instructions and the basal OCR was normalized to the cell number.

### Measurement of metabolites

The culture medium was aspirated from a 10-cm cell culture dish and the cells were washed twice with 5 % mannitol solution (10 mL, followed by 2 mL). The cells were then treated with 800 μL methanol and left at rest for 30 s in order to inactivate enzymes. Next, the cell extract was treated with 550 μL Milli-Q water containing internal standards (H3304-1002, Human Metabolome Technologies, Inc., Tsuruoka, Japan) and left at rest for another 30 s. The extract was obtained and centrifuged at 2300 × *g* and 4 °C for 5 min; 800 μL of the upper aqueous layer was then centrifugally filtered through a Millipore 5-kDa cutoff filter at 9100 × *g* and 4 °C for 120 min to remove proteins. The filtrate was centrifugally concentrated and resuspended in 50 μL of Milli-Q water for capillary electrophoresis time-of-flight mass spectrometry (CE-TOFMS) analysis. The ratios and *P*-values (*t*-test) were calculated by comparing cells transfected with control shRNA (*n* = 3) with those transfected with AK4 shRNA (*n* = 3), or cells transfected with control vector (*n* = 3) versus those overexpressing AK4 (*n* = 3). The data were normalized to the total cell numbers.

### Electron microscopy

Cells cultured on plastic were fixed using a 2 % paraformaldehyde and 2 % glutaraldehyde mixture in 0.1 M cacodylate buffer, pH 7.4, for 30 min and post-fixed in 2 % osmic acid for 1 h. The sheets were dehydrated in graded acetone and embedded in Epon 812. Cells were cut horizontally by an ultramicrotome (Reichert Ultracut) into approximately 90-nm sections. These ultrathin sections were stained using uranyl and lead citrate and examined using a Hitachi H7 ultramicroscope. The mitochondrial diameter was measured by calculating an average of 10 electron microscopy pictures taken at × 10,000 magnification.

### Xenograft models

Ten week-old female nude mice were used for xenografting. The mice were randomly divided into four groups. A HeLa cell/Matrigel mixture (0.2 mL) was injected into the dorsal subcutis of the nude mice. Two groups ofmice were treated with CDDP (5 mg/kg intraperitoneally, once a week). Tumor volume was calculated as follows: volume = 0.5 × length × width^2^.

### Mitochondrial DNA (mtDNA) measurement

mtDNA content was measured by real-time polymerase chain reaction (RT-PCR) with the Human Mitochondrial DNA (mtDNA) Monitoring Primer Set (Takara Japan). Briefly, we quantified mtDNA transcripts (NADH dehydrogenase subunit 1 (*ND1*) and NADH dehydrogenase subunit 5(*ND5*)), and normalized these to nuclear DNA transcripts (Solute Carrier 2B1(*SLCO2B1*) and Serpin peptidase inhibitor, clade A, member 1 (*SERPINA1*)).

### Statistics

Data correspond to the mean ± standard deviation. Unpaired Student’s *t*-tests were used to compare study groups. Analysis of variance (ANOVA) with post-hoc analysis using Turkey’s multiple comparison test was used for comparisons between multiple groups. *P* values < 0.05 were considered to be statistically significant.

## Results

### AK4 expression was up-regulated by hypoxia and DFO

**Fig. 1:**
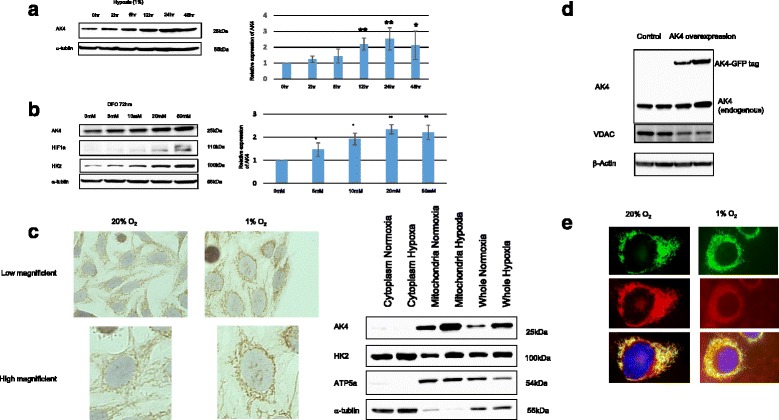
Evaluation of adenylate kinase 4 (AK4) expression and intracellular localization in HeLa cells. **a** Left panel: western blotting evaluation of AK4 (25 kDa) and alpha-tubulin (55 kDa) expression at the indicated time-points under hypoxic conditions (1 % O_2_). Right panel: band densities were quantified using Image Lab (BioRad) and normalized to the internal control (alpha-tubulin). *P < 0.05, **P < 0.01. **b** Left panel: western blotting analysis of AK4 expression after deferoxamine (DFO) treatment. Right panel: quantified data normalized by internal control alpha-tubulin were conducted by using Image Lab (BioRad). *P < 0.05, **P < 0.01. **c** Left panel: immunohistochemical evaluation of the intracellular localization of AK4. Upper panel: normoxia; lower: hypoxia (1 % Day 2); right panel: subcellular localization of AK4. HK2 (100 kDa) was an hypoxic marker, ATP5a (54 kDa) was a mitochondrial marker, and alpha-tubulin (55 kDa) was the loading control. **d** AK4 expression in a stable transformant containing AK4 tagged with GFP on its C terminus. In addition to internal AK4 (25 kDa), overexpressed AK4:GFP (50 kDa) signaling was also detected. VDAC or β-Actin is used for mitochondrial loading control or total protein loading control, respectively. **e** Intracellular localization of the AK4GFP stable transformant. Top: GFP, center: MitoTracker Red, bottom: merged

There are several reports on the regulation of AK4 expression. Hypoxia was previously reported to up-regulate AK4 expression in SHSY5Y and HEK293 cells, but to down-regulate AK4 expression in HepG2 cells [16]. Hepatotoxicans such as acetaminophen, amiodarone, tetracycline, and carbon tetrachloride have been reported to induce AK4 expression [13]. We first compared the effects of these compounds with that of hypoxia and found that hypoxia was the most efficient inducer of AK4 in HeLa cells (data not shown). Therefore, in the present study we focused on the effect of hypoxia to characterize AK4 expression in HeLa cells. When these cells were cultured in 1 % O_2_, increased AK4 protein expression was significantly observed from 12 h onwards (Fig. 1a). We then applied an iron chelator (DFO) to the culture for 72 h, which mimics hypoxia and is known to induce HIF1α accumulation [19]. We observed that AK4 expression was increased in a DFO dose-dependent manner (Fig. 1b). We also detected DFO could increase the expression of HIF1α and HK2 in the same way. Furthermore, we analyzed the AK4 expression in A549 cells, a human alveolar adenocarcinoma cell line, and found that AK4 expression was increased by hypoxia and DFO (Additional file 1: Figure S1A and B). Our previous study indicated that both AK3 and AK4 were co-localized in the mitochondrial matrix under normoxic conditions [20]. However, the localization of AK4 under hypoxic conditions has not been clarified. Therefore, we analyzed the localization of AK4 under both normoxic and hypoxic conditions. Immunohistochemical staining revealed that AK4 was localized within the mitochondria in HeLa cells cultured under 21 % O_2_ (Fig. 1c, upper left panel). After culturing under 1 % O_2_ for 2 days, an increased AK4 signals within mitochondria around the nucleus were detected, but there was no evident translocation of AK4 to outside the mitochondria (Fig. 1c, lower left panel). To confirm it, we examined the subcellular localization of AK4 with western blotting using subcellular fractionated samples (Fig. 1c, right panel). AK4 was specifically detected in the mitochondrial fraction as shown in the positive control, ATP5a, which is one of ATP synthase subunits and a mitochondrial marker, but not as hexokinase 2 (HK2), which associates with both mitochondrial and cytoplasmic compartments, while HK2 expression was enhanced by hypoxia. We then produced a stable cell line over-expressing AK4 tagged with green fluorescent protein (GFP) on its C terminus (AK4GFP) at a level equivalent to that of endogenous AK4 (Fig. 1d). The GFP-tagged AK4 localized around nuclear and merged image with MitoTracker Red indicated that AK4 localizes in mitochondria (Fig. 1e).

### AK4 knockdown increased sensitivity to anti-cancer drug

**Fig. 2:**
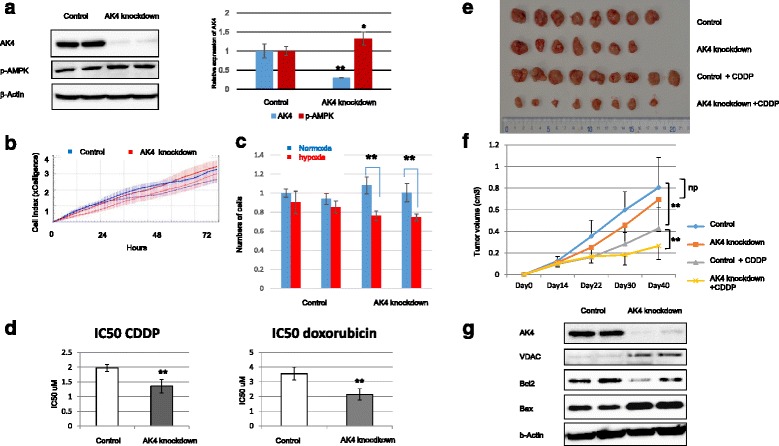
Decreased drug tolerance due to adenylate kinase 4 (AK4) knockdown in HeLa cells. **a** Left panel: western immunoblotting showing AK4 knockdown in cells expressing AK4 short hairpin (sh)RNA. AK4, 25 kDa; phosphorylated 5΄ AMP-activated protein kinase (p-AMPK), 64 kDa; β-actin, 42 kDa. Right panel: band densities were quantified using Image Lab (BioRad) and normalized to the internal control, alpha-tubulin *P < 0.05, **P < 0.01. **b** proliferation curves for AK4 shRNA cells and control shRNA cells, evaluated by xCELLigence (Real Time Cell Analysis System). Cells were evaluated after seeding at 5000 cells/well. The data are shown as mean ± standard deviation (SD) Cell Index (CI) values. **c** Cell numbers were counted 3 days after seeding under normoxic and hypoxic (1 % O_2_) conditions *P < 0.05, **P < 0.01. **d** Evaluation of drug sensitivity after AK4 knockdown showing the half maximal inhibitory concentration (IC50) for cis-diamminedichloro-platinum(II) (CDDP) and doxorubicin. IC50 values were determined by MTS assays. **P < 0.01. **e** Images of tumors resulting from HeLa cells grafted subcutaneously in nude mice on day 40 after grafting. **f** Graph showing the volume of HeLa cell grafts in nude mice at the indicated time-points. The data are shown as mean tumor volume (cm^3^) ± SD, **P < 0.01. **g** Western blotting analysis of HeLa cells 3 days after siRNA treatment. AK4, VDAC, Bax, or Bcl2 were detected at 25 kDa, 39 kDa, 21 kDa, or 26 kDa, respectively

For loss of function analysis, we used AK4 shRNA to produce a stable knockdown of AK4 expression. Western blot analysis demonstrated that transfection of AK4 shRNA effectively down-regulated AK4 protein levels (Fig. 2a). Interestingly, the phosphorylation level of AMPK, was significantly elevated, suggesting the increase of AMP/ATP ratio (Fig. 2a). This finding was consistent with A549 cell line (Additional file 1: Figure S1C) and a previous report [17]. However, no obvious differences in cell proliferation were observed between the AK4 knockdown cells and control cells (Fig. 2b). To further examine whether AK4 is necessary for hypoxic acclimation, we analyzed cell growth under hypoxic conditions. AK4 knockdown cells showed significantly reduced proliferation under hypoxia compared to normoxia, indicating that AK4 may play a role in hypoxic adaptation (Fig. 2c).

As one of characteristic phenotypes in cancer cells, hypoxia-resistant metabolism is recognized to confer increased resistance to apoptosis and to chemotherapy [21]. Therefore, we next examined the drug sensitivity of AK4 knockdown cells. The half maximal inhibitory concentrations (IC50) for CDDP were 1.97 ± 0.12 μM or 1.35 ± 0.22 μM in the cells expressing either control shRNA or AK4 shRNA, respectively. For doxorubicin, the values were 3.55 ± 0.44 μM or 2.15 ± 0.38 μM in them (Fig. 2d). The results clearly indicated that the level of AK4 expression was inversely correlated with drug sensitivity. These findings were consistent with those of A549 cell line (Additional file 1: Figure S1D and E). To confirm the influence of AK4 on drug sensitivity in vivo, we subcutaneously grafted AK4 knockdown cells in matrigel into nude mice. After 40 days, we measured the tumor volume. Tumor volume composed of HeLa cells transfected with AK4 shRNA showed smaller (53.2 %) compared with those transfected with control shRNA. When CDDP was administered into mice, the inhibition of tumor growth was greater for AK4 knockdown cells (62.0 % of the volume of untreated AK4 knockdown cells) than for control cells (86.3 % of the volume of untreated control shRNA cells), indicating that down-regulation of AK4 expression increased the sensitivity of these cells to CDDP (Fig. 2e and f). To further analyze the drug sensitivity, we examined Western blot analysis. The expression level of Bax was increased and that of Bcl2 was decreased in AK4 knockout cells (Fig. 2g).

### AK4 knockdown induces mitochondrial activation

**Fig. 3:**
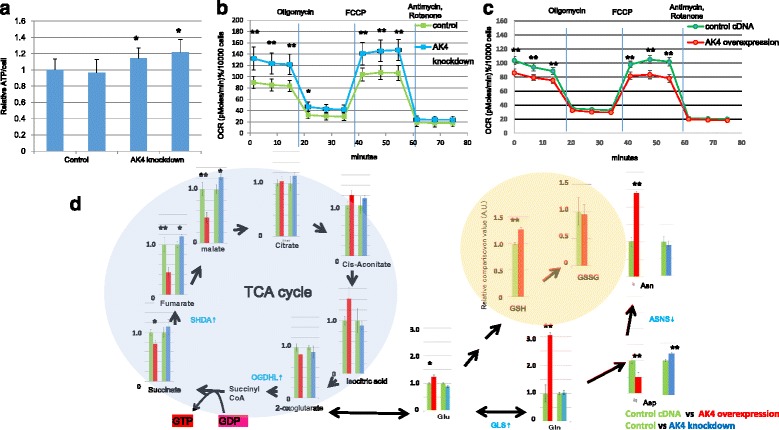
Oxygen consumption rate (OCR) and metabolite analysis in HeLa cells expressing adenylate kinase 4 (AK4) or control short hairpin (sh)RNA. **a** Relative ATP level per cell. 10,000 cells were seeded in 96 wells, and measured 3 days after siRNA treatment. ATP level was normalized by cell number deduced from cyquant assay. *p < 0.05. **b** Flux analyzer OCR measurements were performed in triplicate in cells harboring AK4 shRNA or control shRNA before and after administration of oligomycin, carbonylcyanide-p-trifluoromethoxyphenylhydrazone (FCCP), antimycin, and rotenone, as indicated. *P < 0.05, **P < 0.01. **c** OCR of AK4-overexpressing and control cells before and after administration of oligomycin, FCCP, antimycin, and rotenone, as indicated. **d** Representative metabolomics analysis of compounds involved in the tricarboxylic acid (TCA) cycle. Left: comparison of control vector (green) and AK4 overexpression (red). Right: comparison of control shRNA (green) and AK4 shRNA (blue). *P < 0.05, **P < 0.01

Previous studies demonstrated that siRNA-induced AK4 knockdown increased the amount of ATP per cell [17]. Consistent with this, in the present study we also found that down-regulation of AK4 protein expression by siRNA was associated with increased cellular ATP (Fig. 3a). In order to examine whether mitochondrial activation was involved in the increase of ATP level, we measured the OCR using a flux analyzer using stable AK4 shRNA-expressing knockdown cells. Flux analysis revealed that both basal and maximal respiration rates were increased in AK4 shRNA-expressing HeLa cells (Fig. 3b). On the other hand, we found that both basal and maximal OCR levels were significantly decreased in AK4-overexpressing cells (Fig. 3c). These results indicated that the levels of mitochondrial oxidation are inversely correlated with the level of AK4 expression. To further investigate the effect of the AK4 expression level on cellular metabolites, we performed the metabolome analysis with CE-TOFMS. As shown in Table 1, significant different levels of metabolites were observed in AK4 knockdown cells compared to control cells. The significant difference was also observed in AK4-overexpressing cells compared to control cells as shown in Table 2. In AK4-knockdown cells, down-regulation of NADPH and up-regulation of NADP^+^ were significantly detected, reflecting oxidative stress condition [22]. For the mitochondrial metabolites in tricarboxylic acid (TCA) cycle, there was a significant up-regulation of fumarate and malate in AK4-knockdown cells, and significant down-regulation of succinate, fumarate, and malate in AK4-overexpressing cells. Glutamate was also significantly down-regulated in AK4-knockdown cells, and Glutamate, Glutamine and glutathione were significantly up-regulated in AK4-overexpressing cells (Fig. 3d).

**Table 1:** Metabolic analysis (AK4 shRNA vs control shRNA)

Metabolite	KEGG ID	Ratio	*P*-value
N-Acetylglucosamine 6-phosphate	C00357	1.95	0.006
NADP+	C00006	1.8	0.003
XA0013	No ID	1.72	0.038
UDP-glucuronic acid	C00167	1.6	0.001
Inosine	C00294	1.6	0.185
CoA_divalent	C00010	1.55	0.005
N-Acetylcysteine	C06809	1.53	0.012
Fructose 1,6-bisphosphate	C00354	1.53	0.003
Cysteine	C00097, C00736, C00793	1.52	0.004
Butyrylcarnitine	C02862	1.51	0.011
N-Acetylglucosamine 1-phosphate	C04256	1.49	0.002
1-Methyl-4-imidazoleacetic acid	C05828	1.46	0.038
UDP-N-acetylglucosamine	C00043	1.44	0.001
Rhein	C10401	1.43	0.044
4-Guanidinobutyric acid	C01035	1.41	0.005
1H-Imidazole-4-propionic acid	No ID	1.41	0.031
Alanine	C00041, C00133, C01401	1.38	0.001
XC0016	No ID	1.38	0.001
ADP-glucose GDP-fucose	C00498, C00325	1.34	0.013
Creatine	C00300	1.31	0.011
UDP-glucose UDP-galactose	C00029, C00052	1.31	0.001
dTDP-glucose	C00842	1.3	0.014
Adenosine 5΄-phosphosulfate	C00224	1.29	0.041
Argininosuccinic acid	C03406	1.26	0.008
Myo-inositol 2-phosphate	No ID	1.26	0.006
Glucose 1-phosphate	C00103	1.25	0.025
Pantothenic acid	C00864	1.24	0.022
Isethionic acid	C05123	1.23	0.129
Malic acid	C00149, C00497, C00711	1.23	0.02
CMP-N-acetylneuraminate	C00128	1.23	0.013
Hippuric acid	C01586	1.22	0.098
XA0033	No ID	1.22	0.022
Phosphocreatine	C02305	1.2	0.324
CTP	C00063	1.2	0.021
N-Formylmethionine	C03145	1.2	0.012
S-Adenosylmethionine	C00019	1.2	0.015
GTP	C00044	1.2	0.009
Cys-Gly	C01419	1.2	0.34
2-Aminobutyric acid	C02261, C02356	1.19	0.015
Hydroxyproline	C01157	1.19	0.021
ATP	C00002	1.19	0.001
Threonic acid	C01620	1.18	0.03
N-Acetylaspartic acid	C01042	1.17	0.007
Asparate	C00049, C00402, C16433	1.16	0.009
Fumaric acid	C00122	1.16	0.027
N-Acetylalanine	No ID	1.16	0.049
UTP	C00075	1.14	0.007
Threonine	C00188, C00820	1.11	0.002
Homocitrulline	C02427	1.07	0.041
Betaine	C00719	0.91	0.034
Glutamine	C00025, C00217, C00302	0.89	0.009
1-Methylnicotinamide	C02918	0.89	0.049
Proline	C00148, C00763, C16435	0.89	0.041
Arginine	C00062, C00792	0.86	0.042
UDP	C00015	0.86	0.021
dTMP	C00364	0.85	0.024
NMN	C00455	0.84	0.008
Taurine	C00245	0.82	0.011
5-Aminovaleric acid	C00431	0.81	0.001
Isovalerylcarnitine	No ID	0.79	0.013
Gluconic acid	C00257	0.79	0.008
N-Acetylglutamic acid	C00624	0.78	0.006
AMP	C00020	0.73	0.007
Ala-Ala	C00993	0.71	0.36
GABA	C00334	0.71	0.001
Glutathione (GSSG)_divalent	C00127	0.71	0.024
dTDP	C00363	0.7	0.004
XA0012	No ID	0.7	0.001
XA0027	No ID	0.7	0.02
N-Acetylputrescine	C02714	0.7	0.002
Adenosine	C00212	0.68	0.005
Gluconolactone	C00198	0.66	0.036
dADP	C00206	0.65	0.027
XC0132	No ID	0.59	0.002
GMP	C00144	0.58	0.002
γ Glu-Cys	C00669	0.55	0.006
2΄-Deoxyadenosine 5΄-deoxyadenosine	C00559, C05198	0.53	0.024
Acetylcholine	C01996	0.5	0.007
3-Phosphoglyceric acid	C00197	0.44	0.002
Phosphoenolpyruvic acid	C00074	0.42	0.025
NADPH	C00005	0.36	0.044

**Table 2:** Metabolic analysis (AK4 overexpression vs control)

Metabolite	KEGG ID	Ratio	*p*-value
Kynurenine	C00328,C01718	6.73	0.001
Gln	C00064,C00303,C00819	3.06	0.004
Glycerophosphocholine	C00670	2.76	0.003
Thiamine diphosphate	C00068	2.68	0.001
Glucose 1-phosphate	C00103	2.63	0
Terephthalic acid	C06337	2.55	0
PRPP	C00119	2.45	0.016
N-Acetylglutamic acid	C00624	2.42	0.004
Asn	C00152,C01905,C16438	2.37	0
Pelargonic acid	C01601	2.31	0.017
Glycerol	C00116	2.24	0.003
Ser	C00065,C00716,C00740	2.13	0
XA0033	No ID	2.04	0.024
Lauric acid	C02679	2.02	0.007
Phosphocreatine	C02305	2	0.01
dGTP	C00286	2	0.008
Phosphorylcholine	C00588	1.91	0
dCTP	C00458	1.88	0.005
2-Hydroxyglutaric acid	C02630,C01087,C03196	1.87	0.011
N-Acetylputrescine	C02714	1.87	0
Glycerol 3-phosphate	C00093	1.87	0.018
2-Aminobutyric acid	C02261,C02356	1.87	0.004
Isovalerylcarnitine	No ID	1.87	0.001
myo-Inositol 2-phosphate	No ID	1.82	0.001
dATP	C00131	1.75	0.003
Ornithine	C00077,C00515,C01602	1.7	0.003
dADP	C00206	1.67	0.021
3-Hydroxy-3-methylglutaric acid	C03761	1.64	0.001
Putrescine	C00134	1.62	0.021
Arg	C00062,C00792	1.56	0.009
CTP	C00063	1.55	0.034
Rhein	C10401	1.54	0.023
Ala	C00041,C00133,C01401	1.52	0.015
Thr	C00188,C00820	1.48	0.003
ADP	C00008	1.47	0.032
UDP-glucose	C00029	1.46	0.017
UDP-galactose	C00052		
Carnosine	C00386	1.46	0.029
Lys	C00047,C00739,C16440	1.44	0.002
UDP-N-acetylglucosamine	C00043	1.4	0.013
ADP-glucose	C00498	1.4	0.003
GDP-fucose	C00325		
O-Acetylcarnitine	C02571	1.4	0.007
GABA	C00334	1.39	0.001
Thiamine	C00378	1.39	0.019
His	C00135,C00768,C06419	1.38	0.003
1-Methylnicotinamide	C02918	1.38	0.002
Citrulline	C00327	1.36	0.012
dTDP-glucose	C00842	1.35	0.008
Val	C00183,C06417,C16436	1.35	0.003
Leu	C00123,C01570,C16439	1.35	0.003
Gly	C00037	1.33	0.005
Ile	C00407,C06418,C16434	1.32	0.001
N8-Acetylspermidine	C01029	1.29	0.031
Creatinine	C00791	1.29	0.003
Pantothenic acid	C00864	1.28	0.009
Hydroxyproline	C01157	1.27	0.035
Glutathione (GSH)	C00051	1.26	0.001
Phe	C00079,C02057,C02265	1.26	0.007
Met	C00073,C00855,C01733	1.25	0.007
Homoserinelactone	No ID	1.24	0.005
Glu	C00025,C00217,C00302	1.24	0.016
Pyridoxine	C00314	1.24	0.025
Trp	C00078,C00525,C00806	1.22	0.017
Gluconic acid	C00257	1.22	0.037
N-Formylmethionine	C03145	1.21	0.034
Butyrylcarnitine	C02862	0.78	0.008
Succinic acid	C00042	0.76	0.013
Hypotaurine	C00519	0.74	0.022
Creatine	C00300	0.66	0.001
Choline	C00114	0.65	0.013
Ribulose 5-phosphate	C00199,C01101	0.6	0.023
XA0012	No ID	0.6	0.008
Asp	C00049,C00402,C16433	0.54	0.003
dTMP	C00364	0.52	0.03
XC0016	No ID	0.51	0.001
Thiamine phosphate	C01081	0.48	0.038
Malic acid	C00149,C00497,C00711	0.47	0.006
β-Ala	C00099	0.44	0.004
Fumaric acid	C00122	0.44	0.006
XC0061	No ID	0.43	0.017
3'-Dephospho CoA	C00882	0.42	0.034
XA0002	No ID	0.39	0.027
CMP	C00055	0.38	0.019
UMP	C00105	0.38	0.031
NADP+	C00006	0.36	0.001

Since it was speculated that these changes in the metabolite levels might be due to the changes of transcriptional regulation, we performed microarray analysis to confirm the differential gene expression in AK4 shRNA-expressing cells. As shown in Table 3, among the genes varied in gene expression, we found several interesting changes in gene expression that relate to the metabolic changes. The expression of genes encoding two TCA cycle-related enzymes, SDHA and OGDHL, were up-regulated. SDHA is a subunit of complex 2, and catalyzes the oxidation of succinate and ubiquinone to fumarate and ubiquinol. OGDHL is a rate-limiting component of the multi-enzyme OGDH complex; malfunction of this complex is associated with neurodegeneration [23]. OGDH catalyzes the conversion of 2-oxoglutarate to succinyl-CoA and CO_2_ within eukaryotic mitochondria, controlling a rate-regulating TCA cycle step. Increased expression of these enzymes would be anticipated to contribute to an acceleration of the TCA cycle. We also found altered expression of genes encoding some enzymes controlling the flow of TCA metabolites. GLS is the major enzyme catalyzing the conversion of glutamine to glutamate, and GLS2 increases mitochondrial respiration and ATP generation. We found increases in *GLS* and *GLS2* mRNA expression, but there were no significant changes in the levels of glutamate and glutamine. Expression of Transglutaminase (*TGM2*), which encodes a protein involved in cell differentiation, proliferation, and apoptosis, was also up-regulated. No significant changes in the expression of other AK isozymes were observed.

**Table 3:** Microarray analysis data from HeLa cell. Tricarboxylic acid (TCA) cycle genes showing significant up-regulation are shown in red (*p* < 0.05), and those showing significant down-regulation are shown in blue (*p* < 0.05)

GeneSymbol	Compare1_*P*_Value	Compare1_ratio
AK4	8.65E-08	0.083
CKMT1A	6.81E-04	8.532
CKB	9.73E-03	1.386
OGDHL	3.21E-03	2.127
SDHA	2.15E-03	1.337
GLS	1.39E-02	1.395
GLS2	1.92E-02	2.405
SIRT3	2.30E-02	0.820
MFN2	9.38E-04	1.589
CDKN1B	4.04E-02	1.231
YY1	2.95E-02	1.198
ASNS	4.89E-03	0.684
TGM2	1.86E-02	1.276
SLC2A1	8.40E-02	1.286
AK1	9.18E-01	0.992
AK2	7.42E-02	0.853
AK3	3.85E-01	1.066
AK5	6.44E-01	0.908

### AK4 knockdown altered mitochondrial properties

**Fig. 4:**
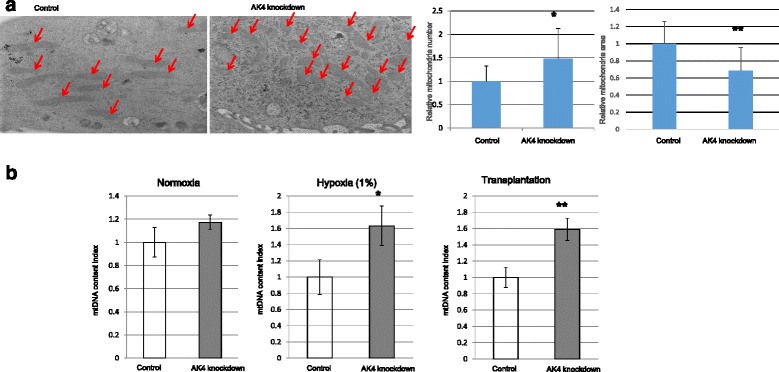
Alteration of mitochondria by adenylate kinase 4 (AK4) knockdown. **a** Representative electron microscope images of cells in normoxic condition. Left panel: HeLa cells transfected with control short hairpin (sh)RNA. Right panel: HeLa cells expressing adenylate kinase 4 (AK4) shRNA. The arrows indicate mitochondria. Right: The graphs show the relative mitochondrial counts and cross-sectional area, as indicated. Mitochondrial numbers were counted in ten random pictures taken at a magnification of 10,000. Mitochondrial size was determined using BZ-II analyzer (Keyence) software. We examined 70 mitochondria in control shRNA cells and 100 mitochondria for in AK4 shRNA cells. **b** Relative mitochondrial (mt)DNA copy number under the conditions indicated. mtDNA was measured by real-time PCR using the Human mtDNA Monitoring Primer Set (Takara Japan). Briefly, we measured mtDNA (*ND1* and *ND5*), then normalized to the nuclear DNA level (*SLCO2B1* and *SERPINA1*). The mtDNA content index was the ratio of mtDNA/nuclear DNA, calculated by dividing the *ND1* and *ND5* signals by the *SLCO2B1* and *SERPINA1* signals. *P* < 0.05, **P < 0.01

To further examine the role of AK4 in mitochondrial function, we investigated the mitochondrial structure in AK4 knockdown cells using electron microscopy. The number of mitochondria per unit surface area of cytoplasm was increased in AK4 shRNA-expressing cells compared to control shRNA-expressing cells (Fig. 4a). Then, to confirm the mitochondrial amounts, we measure the relative mtDNA level by RT-PCR. Under normoxic conditions, the mtDNA level was slightly higher in AK4 shRNA cells (1.17 ± 0.66) than in control shRNA cells (1.00 ± 0.13), although this difference was not statistically significant (*P* = 0.10). After culturing under 1 % O_2_ for 3 days, the mtDNA count was significantly higher in AK4 shRNA cells (1.63 ± 0.24) than in control shRNA cells (1.00 ± 0.21; *P* = 0.028). When implanted in nude mice, the mtDNA level in the tumors derived from AK4 shRNA cells was significantly higher (1.59 ± 0.13) than in those from control shRNA cells (1.00 ± 0.12; *P* < 0.005) (Fig. 4b). These results clearly demonstrated that the mitochondrial numbers are inversely correlated with the AK4 expression level.

## Discussion

Previous study demonstrated that AK4 expression was induced in the mouse liver treated with hepatotoxicns by proteome analysis [13], indicating that AK4 is one of the markers for oxidative stress response. Up-regulation of AK4 expression in cells cultured under hypoxic conditions has also been reported [24], however, the biological role of AK4 remains unclear.

We first verified the manner of AK4 expression in HeLa cells. AK4 expression was up-regulated by hypoxia (Fig. 1a), and DFO treatment which mimics hypoxia and induces the accumulation of HIF1α by inhibiting its hydroxylation (Fig. 1b). Immunohistochmical and subcellular fractionation analyses confirmed that AK4 was specifically induced in mitochondria (Fig. 1c). Based on these in vitro findings using the different set, which were consistent with the previous report [15, 17], we hypothesize that an increase in AK4 expression could enable acclimation to hypoxia and drug resistance by controling mitochondrial activity.

To examine the role of AK4 on cell growth in vivo, we implanted AK4 knockdown HeLa cells into nude mice and analyzed the tumor size. Tumor sizes formed by AK4 knockdown cells are significantly smaller than those by control cells. This finding might reflect the low subcutaneous oxygen concentration, which would reduce the ability of AK4 knockdown cells to proliferate as shown in Fig. 2e. Administration of CDDP inhibited the proliferation of AK4 knockdown cells more strongly than control cells (Fig. 2e and f), suggesting that down-regulation of AK4 expression increased the efficacy of anticancer drugs. Regulation of mitochondrial activity is now one of the important points for better understanding cancer biology [25, 26]. For example, the tumor cells with reduced mitochondrial activity exhibit drug resistance, where glycolysis predominates [27]. Therefore, much attention is now focused on drugs that may induce a metabolic shift from glycolysis (in the cytoplasm) to oxidative phosphorylation (in the mitochondria) in order to develop new cancer therapy [28]. It was shown that increased glycolysis led to hyperpolarization of mitochondria and inhibition of apoptosis through an increase in the hexokinase level [29]. However, restoration of mitochondrial functions could lead to apoptosis of cancer cells concomitant with the suppression of aerobic glycolysis [30]. Therefore, chemical compounds such as sodium dichloroacetate which inhibits pyruvate dehydrogenase kinase and activates mitochondria are applicable to cancer therapy [31, 32]. In this study the expression level of Bax was increased and that of Bcl2 was decreased in AK4 knockout cells, indicating that AK4 knockdown cell is sensitive to drug due to activation of apoptosis. Taken together, it is suggested that the control of AK4 expression may provide a novel anticancer target through regulating mitochondrial activity.

For a role of AK4 expression in mitochondrial activity, we examined the oxygen consumption rate in AK4 knockdown cells and found that it was increased (Fig. 3b), while it was decreased in AK4 overexpression (Fig. 3c). Moreover, metabolome analysis revealed that TCA cycle metabolites such as fumarate and malate were increased in AK4 knockdown cells, and conversely, these metabolites were down-regulated by AK4 overexpression (Fig. 3d). Interestingly, AK4 overexpression cells showed the increased levels of metabolites such as Gln, Asn and GSH, suggesting the increased glutaminolysis and anti-oxidative activity. These findings demonstrated the biological activity of AK4 that could support higher proliferative activity and resistance to anti-tumor drug in vivo.

Lanning et al. identified the changes in nucleotide pools and central carbon metabolites in cells transfected with AK4 siRNA compared with control siRNA, in which the levels of ATP, fructose 1,6-bisphosphatase (FBP), malate, and the citrate/isocitrate ratio were significantly increased in cells transfected with AK4 siRNA, but they did not mention about other metabolites. In the present study, we found that the levels of ATP, FBP, and malate were increased in AK4 knockdown cells (Fig. 3a, Table 1). In connection with this, we found several changes in gene expression that were correlated with the metabolic shift. The expression of genes encoding two TCA cycle-related enzymes, SDHA and OGDHL, were up-regulated in AK4 knockdown cells. SDHA is a subunit of complex 2, and catalyzes the oxidation of succinate and ubiquinone to fumarate and ubiquinol. OGDHL is a rate-limiting component of the multi-enzyme OGDH complex, which catalyzes the conversion of 2-oxoglutarate to succinyl-CoA and CO_2_ within eukaryotic mitochondria. Increased expression of these enzymes would be anticipated to contribute to an acceleration of the TCA cycle.

From the structural viewpoint of mitochondria, electron microscopic analysis [33, 34] revealed that mitochondrial cross-sectional area was increased in AK4 knockdown cells. Real-time PCR analysis confirmed the increased mtDNA amounts in AK4 knockdown cells. These morphological changes in AK4 knockdown cells might reflect the mitochondrial metabolic activity including TCA cycle and oxidative metabolism as shown by metabolomic and flux analyses. These results are quite important findings because reduction of mitochondrial function and the mtDNA level is associated with tumor progression including distant metastasis, poor survival in early-stage laryngeal cancer, tumor invasion depth, increased tolerance of hypoxia, and TMN stage [35].

**Fig. 5:**
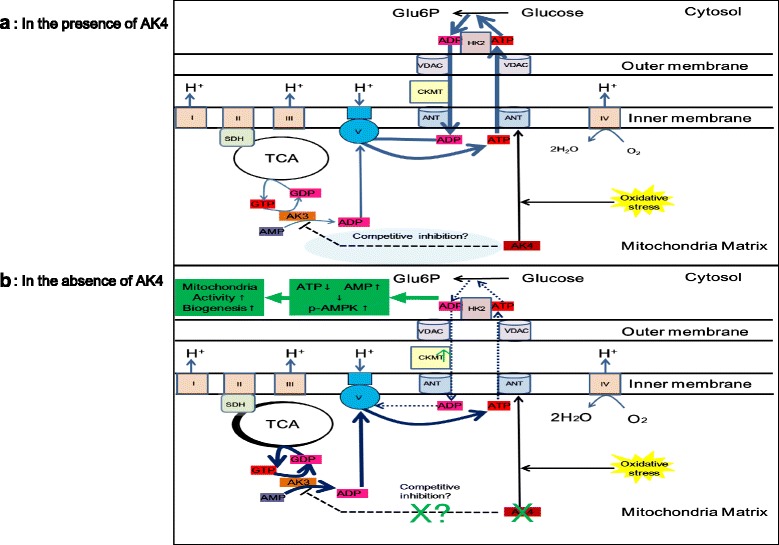
A proposed role of adenylate kinase 4 (AK4) in mitochondria. **a** Proposed adenine nucleotide metabolism in the presence of AK4. ADP that returns to the mitochondria is converted to ATP by ATP synthase. AK4 forms complexes with hexokinase 2 (HK2), voltage-dependent anion channel (VDAC), and adenine nucleotide translocase (ANT) for the efficient recycling of ADP. This interaction with the mitochondrial inner membrane protein, ANT, is important for AK4-mediated protection from oxidative stress. AK3 is thought to be important for regeneration of GDP, which is necessary for the tricarboxylic acid (TCA) cycle. I, II, III, IV, and V indicate complexes I, II, III, IV and V (ATP synthase), respectively. AK4 may also cause competitive inhibition of AK3, which recycles the GDP required for the TCA cycle. **b** Proposed adenine nucleotide metabolism in the absence of AK4. HK2, VDAC, and ANT do not recycle ADP efficiently in the absence of AK4. The ATP concentration decreases focally in the cytosol, leading to 5΄ AMP-activated protein kinase (AMPK) phosphorylation and mitochondrial activation. AK4 knockdown may promote AK3 activity by canceling its inhibition to AK3. This proposed hypothesis remains to be tested. Dotted lines indicate reduced efficiency of ADP recycling

Based on the findings, we propose a working model of the role of AK4 in the regulation of mitochondrial activity (Fig. 5). Hypoxia increases the expression of AK4, which forms the complex with HK2, VDAC, and ANT to increase ADP recycling (Fig. 5a). The ADP that is efficiently back to the mitochondrion is converted to ATP by ATP synthase. ATP is consumed as a substrate for HK2 to enhance glycolysis. A previous study demonstrated that the interaction with the inner mitochondrial membrane protein, ANT, is important for AK4-mediated protection against oxidative stress [11]. However, in the absence of AK4 expression (Fig. 5b), ATP production is increased by mitochondrial activation. This may involve AMPK, a cellular energy sensor that monitors the AMP level. When the cellular ATP level is decreased, the AMP level is increased, resulting in triggering AMPK phosphorylation and activation as previously demonstrated [17]. Activation of AMPK induces mitochondrial biogenesis [36]. As shown in Fig. 2a, we observed up-regulation of p-AMPK in AK4 knockdown cells, suggesting an increase of cytoplasmic AMP. This AMP increase may be caused by a reduced efficiency of ATP transport out of the mitochondria, since it is suggested that AK4 is required for forming the functional complexes with VDAC, ANT, and CypF for efficient ADP recycling [12]. Microarray data showed an increase of *CKMT1* expression (Table 3), which is known to form complexes with VDAC, ANT and other substances in the mitochondrial intermembrane space [37], suggesting a compensational response to insufficient ADP recycling caused by AK4 knockdown. According to the results, it is supported that raised cellular AMP might activate AMPK and increase p-AMPK, resulting in an increase in ATP through promoting mitochondrial activation and biogenesis.

AK4 knockdown produced gross changes in the levels of fumarate and malate in the TCA cycle, possibly due to up-regulated expression of GLS2, a mitochondrial glutaminase, which catalyzes the hydrolysis of glutamine to glutamate and ammonia (Table 3). It is interesting to note that glutamine not only enters the TCA cycle via the conversion of glutamate to α-ketoglutarate, but also maintains the NADPH pool and may act as a precursor for *de novo* pyrimidine synthesis [38]. Another possible mechanism underlying these effects of AK4 is the competitive inhibition of AK3. Both AK3 and AK4 have highly homologous sequences and are found in the mitochondrial matrix, and AK3 is believed to be involved in supplying GDP, which is required for the conversion of succinyl-CoA to succinate. Because AK4 is able to bind nucleotide, substrate competition with AK3 may occur. If competitive inhibition by AK4 reduces AK3 activity and the supply of GDP for the TCA cycle is interrupted, AK4 would impede the reactions downstream from succinate and thus increase glutamate levels. Metabolome analysis demonstrated significant down-regulation of succinate, fumarate, and malate and up-regulation of glutamine and glutamate, consistent with this scenario. However, further analysis is required to confirm the metabolic role of AK4.

## Conclusion

In conclusion, our findings indicated that the AK4 expression level could modulate the anti-cancer drug sensitivity through regulating mitochondrial activity. Control of AK4 expression may provide a novel anticancer therapeutic target.

## Additional file

Additional file 1: Supplemenal Fig. 1 Evaluation of adenylate kinase 4 (AK4) in A549 cells. (A) Western blotting evaluation of AK4 (25 kDa) and α-tubulin (55 kDa) expression at the indicated time-points under hypoxic conditions (1 % O_2_). (B) Western blotting analysis of AK4 expression after deferoxamine (DFO) treatment. (C) Western immunoblotting showing AK4 knockdown in cells by siRNA. AK4, 25 kDa; phosphorylated 5΄ AMP-activated protein kinase (p-AMPK), 64 kDa; β-actin, 42 kDa. (D) Cell numbers were counted 2 days after seeding under either normoxic or hypoxic (1 % O_2_) conditions. **P* < 0.05, ***P* < 0.01. (E) Evaluation of drug sensitivity after AK4 knockdown showing the half maximal inhibitory concentration (IC50) for cis-diamminedichloro-platinum(II) (CDDP). (Upper) IC50 curves of CDDP at 24 h, (Lower) IC50 of CDDP at 24 h. ***P* < 0.01 (PDF 246 kb)

## Abbreviations

AK: adenylate kinase
ANT: adenine nucleotide translocase
CDDP: *cis*-diamminedichloro-platinum (II)
CE-TOFMS: capillary electrophoresis time-of-flight mass spectrometry
DFO: deferoxamine
FCCP: carbonylcyanide-*p*-trifluoromethoxyphenylhydrazone
GFP: a green fluorescent protein
HIF1α: hypoxia inducible factor 1α
HK2: hexokinase 2
mtDNA: mitochondrial DNA
OCR: oxygen consumption rate
OGDHL: 2-oxoglutarate dehydrogenase complex component E1-like
p-AMPK: phosphorylated 5΄ AMP-activated protein kinase
RNAi: RNA interference
SDHA: succinate dehydrogenase complex subunit A
shRNA: short hairpin RNA
siRNA: small interfering RNA
TG: Transglutaminase
VDAC: voltage-dependent anion channel
