# Supplementary figures and images for: Modulation of anti-cancer drug sensitivity through the regulation of mitochondrial activity by adenylate kinase 4
Source: J Exp Clin Cancer Res. 2016 Mar 16;35:48. doi: 10.1186/s13046-016-0322-2 (PMC4793738; doi:10.1186/s13046-016-0322-2)

Supplementary

A

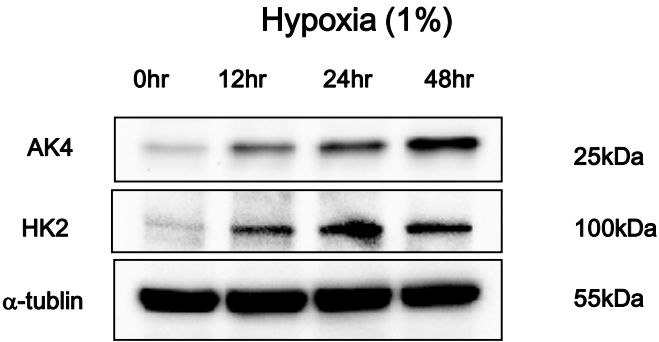

B

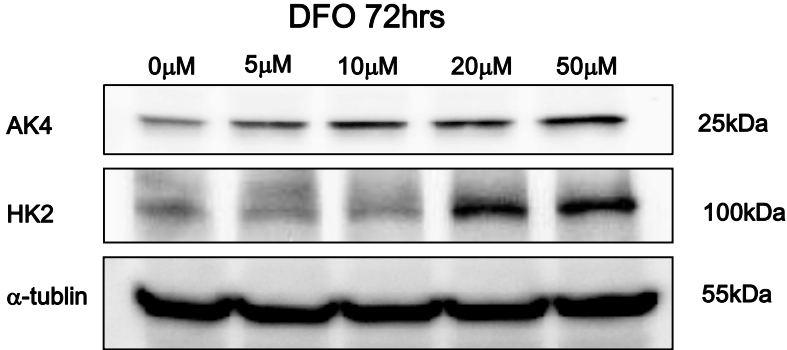

C

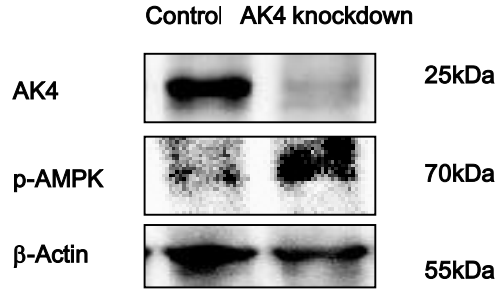

D

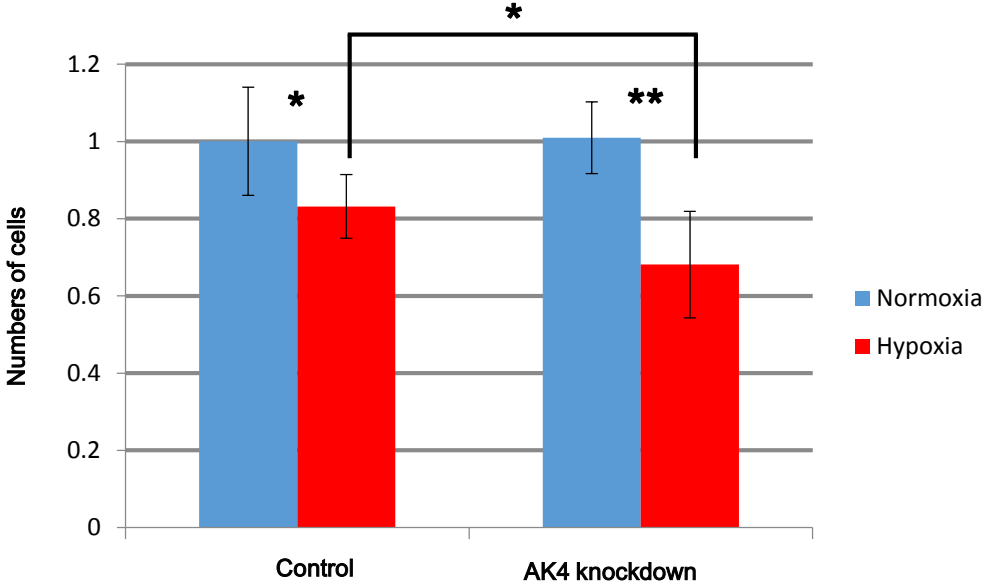

E

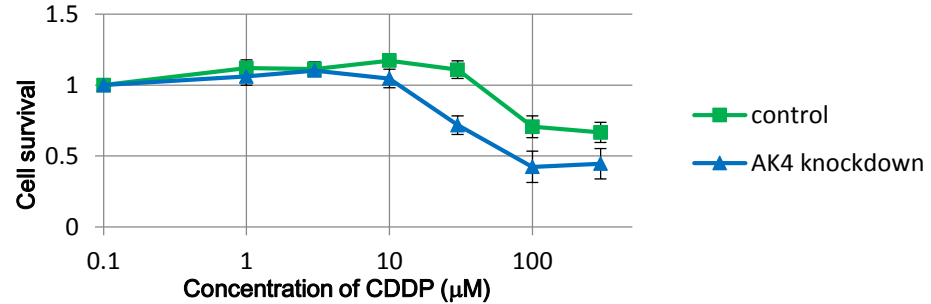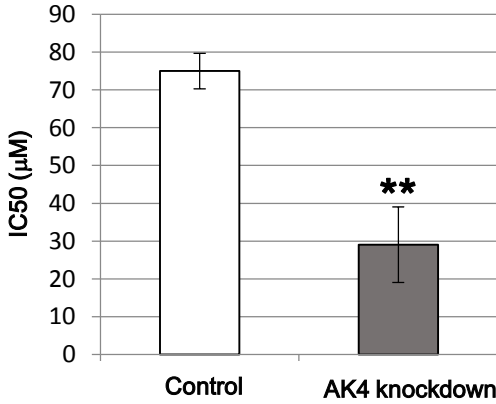

Supplement: Additional file 1: — Supplemenal Fig. 1 Evaluation of adenylate kinase 4 (AK4) in A549 cells. (A) Western blotting evaluation of AK4 (25 kDa) and α-tubulin (55 kDa) expression at the indicated time-points under hypoxic conditions (1 % O2). (B) Western blotting analysis of AK4 expression after deferoxamine (DFO) treatment. (C) Western immunoblotting showing AK4 knockdown in cells by siRNA. AK4, 25 kDa; phosphorylated 5΄ AMP-activated protein kinase (p-AMPK), 64 kDa; β-actin, 42 kDa. (D) Cell numbers were counted 2 days after seeding under either normoxic or hypoxic (1 % O2) conditions. *P < 0.05, **P < 0.01. (E) Evaluation of drug sensitivity after AK4 knockdown showing the half maximal inhibitory concentration (IC50) for cis-diamminedichloro-platinum(II) (CDDP). (Upper) IC50 curves of CDDP at 24 h, (Lower) IC50 of CDDP at 24 h. **P < 0.01 (PDF 246 kb) [file 13046_2016_322_MOESM1_ESM.pdf]
